# Photodynamic Therapy with Natural Photosensitizers in the Management of Periodontal Disease Induced in Rats

**DOI:** 10.3390/gels8020134

**Published:** 2022-02-20

**Authors:** Laura Monica Dascalu (Rusu), Marioara Moldovan, Codruta Sarosi, Sorina Sava, Alexandra Dreanca, Calin Repciuc, Robert Purdoiu, Andras Nagy, Mîndra Eugenia Badea, Ariadna Georgiana Paun, Iulia Clara Badea, Radu Chifor

**Affiliations:** 1 Department of Prosthodontics and Dental Materials, Iuliu Hatieganu University of Medicine and Pharmacy, 31 Avram Iancu Str., 400083 Cluj-Napoca, Romania; dascalu.monica@umfcluj.ro; 2 Raluca Ripan Institute of Chemistry, Babes-Bolyai University, 30 Fantanele Str., 400294 Cluj-Napoca, Romania; marioara.moldovan@ubbcluj.ro; 3 Pathophysiology/Toxicology Department, Faculty of Veterinary Medicine, University of Agricultural Science and Veterinary Medicine, 3-5 Calea Manastur, 400372 Cluj-Napoca, Romania; alexandradreanca@gmail.com (A.D.); calin_c_repciuc@yahoo.com (C.R.); robert.purdoiu@usamvcluj.ro (R.P.); nagyandras26@gmail.com (A.N.); 4 Department of Preventive Dental Medicine, Iuliu Hatieganu University of Medicine and Pharmacy, 31 Avram Iancu Str., 400083 Cluj-Napoca, Romania; mebadea@umfcluj.ro (M.E.B.); iulia.badea@umfcluj.ro (I.C.B.); chifor.radu@umfcluj.ro (R.C.); 5 Department Community Medicine, Iuliu Hațieganu University of Medicine and Pharmacy, 31 Avram Iancu Str., 400083 Cluj-Napoca, Romania; badea.ariadna@umfcluj.ro

**Keywords:** gels, photosensitizers, photodynamic therapy, cytotoxicity, computed tomography

## Abstract

This study aims to investigate the effect of new natural photosensitizers (PS) (based on oregano essential oil, curcuma extract, and arnica oil) through in vitro cytotoxicity and biological tests in rat-induced periodontal disease, treated with photodynamic therapy (aPDT). The cytotoxicity of PS was performed on human dental pulp mesenchymal stem cells (dMSCs) and human keratinocyte (HaCaT) cell lines. Periodontal disease was induced by ligation of the first mandibular molar of 25 rats, which were divided into 5 groups: control group, periodontitis group, Curcuma and aPDT-treated group, oregano and aPDT-treated group, and aPDT group. The animals were euthanized after 4 weeks of study. Computed tomography imaging has been used to evaluate alveolar bone loss. Hematological and histological evaluation showed a greater magnitude of the inflammatory response and severe destruction of the periodontal ligaments in the untreated group.. For the group with the induced periodontitis and treated with natural photosensitizers, the aPDT improved the results; this therapy could be an important adjuvant treatment. The obtained results of these preliminary studies encourage us to continue the research of periodontitis treated with natural photosensitizers activated by photodynamic therapy.

## 1. Introduction

There are a number of oral diseases that pose a major threat to public health worldwide, among which periodontal diseases and dental caries are the most invasive and difficult to treat. The need to develop an alternative prevention treatment with antibacterial agents is due to the side effects of conventionally used treatments (antibacterial, anti-inflammatory, or antibiotic drugs) and the increasing bacterial resistance to them. Thus, natural products and photodynamic therapy has become a topical and important research subject.

Periodontitis is a very common chronic inflammatory disease nowadays. It is caused by infection with the presence of various types of oral bacteria, the so-called “red complex”, which includes, among others, Porphyromonas gingivalis, Tannerella forsythia, Prevotella intermedia as well as other Gram-negative anaerobes wich are organized as dental biofilm [1,2,3].

For periodontal disease to occur, bacteria should be able to colonize the subgingival space and promote the appearance of virulent factors that could eventually affect the host tissue. There are several studies in the literature that have concluded that microbial etiology of periodontal disease has been attributed to a varied number of bacteria organized in biofilm and not just a single microorganism [4,5,6]. 

Conventional mechanical debridement (scaling and root planing) can achieve a temporary decrease in the subgingival levels of pathogens. However, organisms cannot be removed from the majority of periodontal pockets by mechanical therapy alone. In addition, various systemic and local chemical antimicrobial agents (chlorhexidine, enamel matrix derivative, and hyaluronic acid) have been introduced for the treatment of periodontitis, which suppress periodontal pathogens with greater efficacy than mechanical techniques and improve the results of conventional mechanical therapeutic techniques. Some disadvantages of antimicrobial agents’ usage (such as antibiotics) include antibiotic resistance, immune suppression, and other unfavorable reactions. Considering the complications above, it is necessary to expand research in an attempt to find alternative antimicrobial techniques, such as natural agents for antimicrobial therapy. One of them is the use of lasers and photodynamic therapy, which might be effective in eliminating microbes in local and superficial infections in the presence of natural photosensitizers. The literature presents many studies regarding antimicrobial photodynamic therapy (aPDT). Antimicrobial chemotherapy may reduce periodontal pathogens and enhance the results of conventional mechanical treatment [1,7,8,9,10]. 

Antimicrobial photodynamic therapy is a new and promising alternative that aims to remove or reduce pathogenic microorganisms, both Gram-positive and Gram-negative bacteria, as well as viruses, parasites and fungi. Current research is looking for new approaches that can be bactericidal but also have advantages over traditional antibiotic therapy. PDT is a non-thermal photochemical reaction that requires the co-participation of three factors, namely visible light at an appropriate wavelength, oxygen and a photosensitizer. The properties of PSs have been studied in the literature, including their affinity for binding to the bacterial wall and thus efficiently generating reactive oxygen species (ROS) upon photostimulation. Various studies show that there are different types of ROS generated during aPDT, among which singlet oxygen (1O2) is considered the most potential, which means that it is mainly responsible for photo damage and cytotoxic reactions [7,9,11,12].

Turmeric (isolated from *Curcuma longa* L.), is generally known and used as a spice, but has also been shown to have therapeutic effects. Among them is worth mentioning the therapeutic effects in case of liver diseases, wounds and inflamed joints and it’s also known for its blood purification and its antimicrobial effect. In terms of cytotoxicity, turmeric did not show toxic effects on cell cultures and animal studies. It has a wide absorption range of 300-500 nm (with maximum absorption at 430 nm) and produces strong phototoxic effects, thus being a suitable compound for use as PS. Being a fat-soluble material, this PS has certain restrictions, namely, it requires an oil or other synthetic material to make its water solubility possible, such as for example arnica oil [13].

Oregano essential oil is a volatile compound in which carvacrol, p-cymene, γ-terpinene, and α-humulene have been identified. Like other essential oils, it has antimicrobial and antioxidant properties, and is also able to inhibit the growth of *Escherichia coli* and *Staphylococcus aureus*. The compounds mainly responsible for its antioxidant and antibacterial properties are carvacrol and thymol. Its maximum absorption is at 270 nm [14].

aPDT is a new approach that involves combining a non-toxic PS and a low intensity visible light source. This method of treatment has been shown to have an important antimicrobial effect, so it’s now an alternative for treating biofilm-related diseases. aPDT has been researched as an alternative and promising method for the eradication of oral pathogenic bacteria that over time can lead to endodontic disease, periodontitis, periimplantitis and caries [15,16,17,18,19].

The novelty of this study is the formulation and characterization of new photosensitizers used in photodynamic therapy, based on natural extracts and investigated through biological and cytotoxicity tests. The purpose of the study was to obtain new photosensitizers, using oxygen-enriched water and essential oils, to enhance the biological properties of photodynamic antimicrobial therapy used in the treatment of experimentally induced periodontal disease.

## 2. Results and Discussion

### 2.1. Cytotoxicity

**Figure 1:**
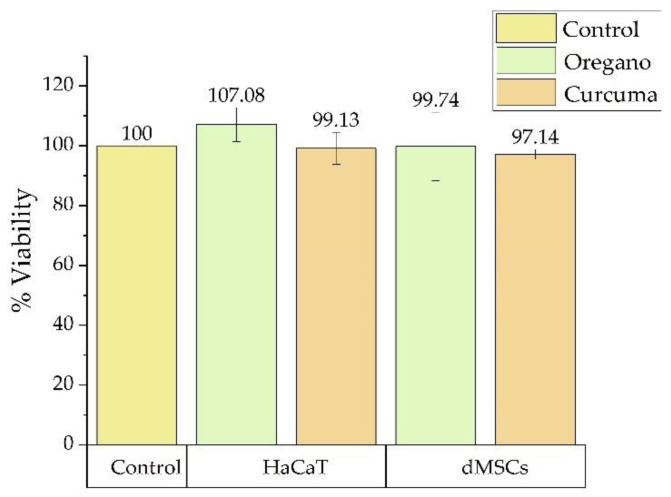
Cell viability of photosensitizers on human dental pulp mesenchymal stem cells (dMSCs) and human keratinocyte cell lines (HaCaT) compared with untreated control. Each bar represents mean and standard deviation (*n* = 3).

Data are shown as the percentage of the average proliferation rate compared to the un-treated control cells. All tests were made three times in exactly the same way and data are presented in Figure 1. The statistic results presents no significant viability differences between the control group and that with experimental gels. As can be seen in Figure 1, the compounds studied had a reduced cytotoxic effect on cell cultures. The most significant decrease in viability was recorded for turmeric gel in human dental pulp mesenchymal stem cells (dMSCs). The toxicity results on the human keratinocyte cell lines (HaCaT) of investigated oregano gel present lower values than curcuma and control gels. 

The ANOVA test had a significant interaction between the treated groups (*p* = 0.40655). Therefore, viability shows that the experimental gels were well tolerated by human dental pulp mesenchymal stem cells and human keratinocyte cell lines, with no signs of toxicity to any of the materials tested.

### 2.2. Clinical Evaluation

Body weight measurements were assessed in order to correlate the clinical status of the animal with the pathology induced. Statistical differences were interpreted (before and after treatment) by using Student *t*-tests. Even though all groups experienced gain in body weight, groups treated with photosensitizers and aPDT showed a more significant degree of weight gain (Table 1).

### 2.3. Computed Tomography (CT) Analysis

**Figure 2:**
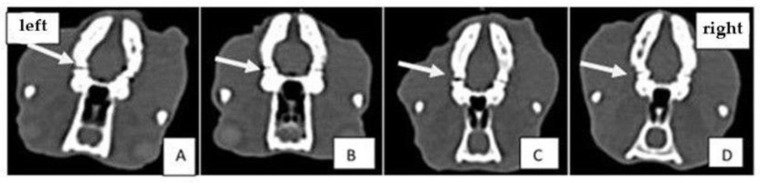
Comparative analysis of CT images of experimental groups on the first day of treatment: (**A**) positive control group; (**B**) aPDT + curcumin group; (**C**) aPDT + oregano group; (**D**) laser therapy group. The arrow indicates the first molar on the left hemimandible where periodontal disease was induced.

**Figure 3:**
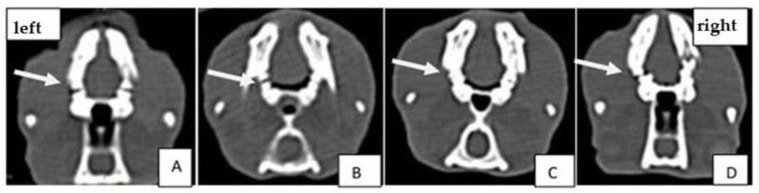
Comparative analysis of CT images of experimental groups on 21st day of treatment: (**A**) positive control group; (**B**) aPDT + curcumin group; (**C**) aPDT + oregano group; (**D**) laser therapy group. The arrow indicates the first molar on the left hemimandibula where periodontal disease was induced.

CT images of experimental groups of Wistar rats on the 1st and 21st day of treatment after the periodontal disease was induced are presented in Figure 2 and Figure 3.

CT scans showed bone loss at the beginning of the experiment when periodontal disease was induced. At the end of the treatment, in the group treated with experimental photosensitizers, an improvement in bone loss and bone density was observed. The analyzed CT images show that after performing the laser treatment in the presence of natural photosensitizers (curcuma or oregano), both values of bone density and periodontal space improved. The groups treated with PS and light showed better results than the group treated with laser only. This demonstrates the treatment-enhancing effect when a PS is combined with laser therapy.

### 2.4. Histological Analysis

**Figure 4:**
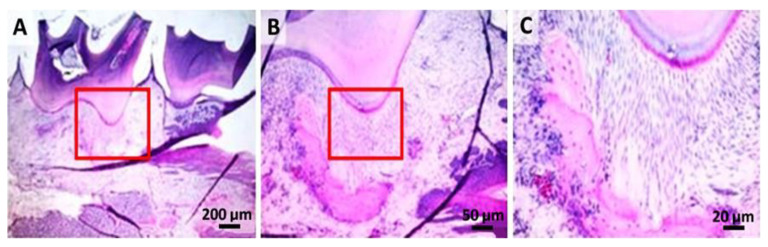
Control group: (**A**) histopathological images from the dental crown and partially from the dental root area (hematoxylin-eosin); (**B**,**C**) higher resolution, 50 µm and 20 µm, of the marked zone with red.

Following the histological analysis, the tooth and the periodontal ligament, respectively, and the dental alveolar bone (the dental support device) showed a normal appearance in the M group (Figure 4).

**Figure 5:**
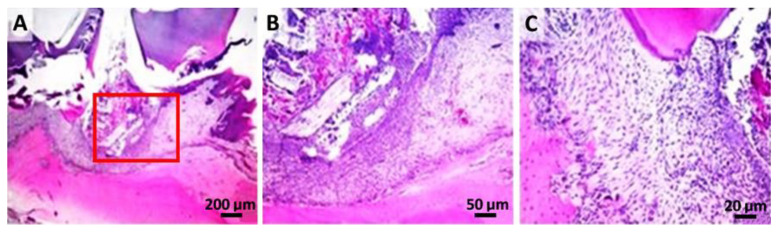
Periodontisis group: (**A**,**B**) the superficial area of the inflammatory outbreak covered by tissue and fodder debris; (**C**) detail of the dental support device, with moderate-segmental osteoclastic resorption of the alveolar bone and suppurated periodontitis; (hematoxylin–eosin).

In the cervical and interdental space, moderate gingival retractions associated with chronic and superficial focal gingivitis were observed (group P). The superficial area of the inflammatory outbreak is covered by an abundant serum–leukocyte crust mixed with tissue and fodder debris. In addition, a moderate-segmental osteoclastic resorption of the alveolar bone was observed combined with suppurative (moderate) periodontitis extending from the previously described gingival defect. At the level of the sub-gingival area, abundant granulation tissue that partially delimits the septic focal point and replaces the dental ligament focal point was observed (Figure 5).

**Figure 6:**
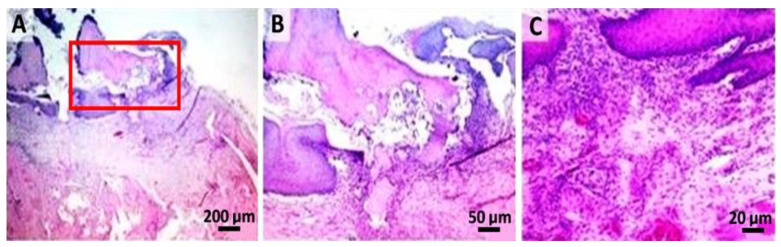
Laser group: (**A**) hyperplasia and marked hyperkeratosis of the gingival epithelium (hematoxylin–eosin); (**B**) high resolution, 50 µm, of the marked zone with red from image A; (**C**) numerous neutrophils with rare mononuclear and reactive fibroblasts.

In the L group (Figure 6), an important hyperplasia and hyperkeratosis (orthokeratotic) of the gingival epithelium with the formation of irregular, anatomic epithelial papillae, separated by a fibro-vascular inflammatory stroma (primarily neutrophils), moderate-segmental osteoclastic resorption of the alveolar bone, and suppurated periodontitis was observed. The inflammatory process was represented by bands and degenerated neutrophils in mixture with rarely mononuclear cells and reactive fibroblasts.

**Figure 7:**
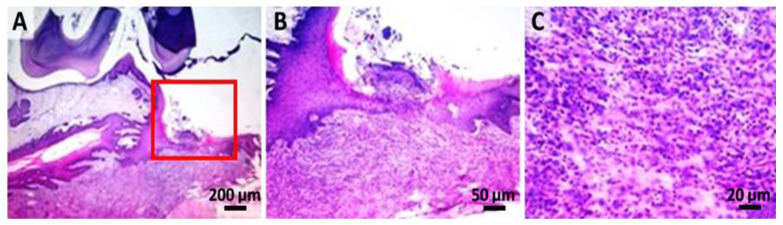
GC group: (**A**) hyperplasia and marked hyperkeratosis of the gingival epithelium. The superficial gingival area presents a focal ulcer (minimal), covered by a serocellular crust mixed with cellular debris and forage (the area demarcated by the rectangle); (**B**) higher resolution, 50 µm, of the marked zone with red; (**C**) detail of inflammatory granulation tissue, with the abundance of neutrophils (viable and degenerate) in combination with rare mononuclear and reactive fibroblasts.

In the GC group (Figure 7), an important hyperplasia and hyperkeratosis (orthokeratosis) in the gingival epithelium was noticed. Therefore, the formation of irregular, anastomosing epithelial papillae, separated by an abundant (inflammatory granulation tissue) fibro-vascular inflammatory stroma (primarily neutrophils and macrophages) was observed. Furthermore, the superficial gingival area presented a focal ulcer (minimal) covered by a serum cellular crust mixed with cellular debris and fodder. In Figure 7C, a detailed aspect of inflammatory granulation tissue with abundant neutrophils (viable and degenerate) mixed with rarely mononuclear cells and reactive fibroblasts were highlighted.

**Figure 8:**
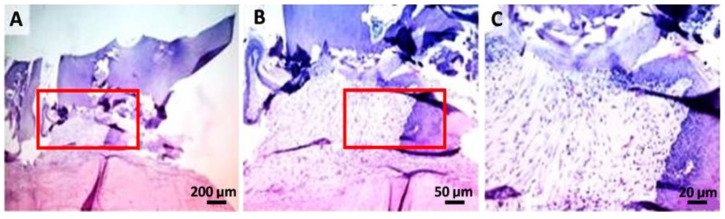
GO group: (**A**,**B**) hyperplasia and marked hyperkeratosis of the gingival epithelium, and partial replacement of the dental ligament with partially oriented fibro-vascularized connective tissue; (**C**) high resolution, 20 µm, of the marked zone with red from image B.

In the GO group, marked gingival epithelial hyperplasia and hyperkeratosis (orthokeratosis) and partial replacement of the dental ligament with partially oriented fibro-vascular connective tissue was observed (Figure 8).

### 2.5. Complete Blood Count

**Figure 9:**
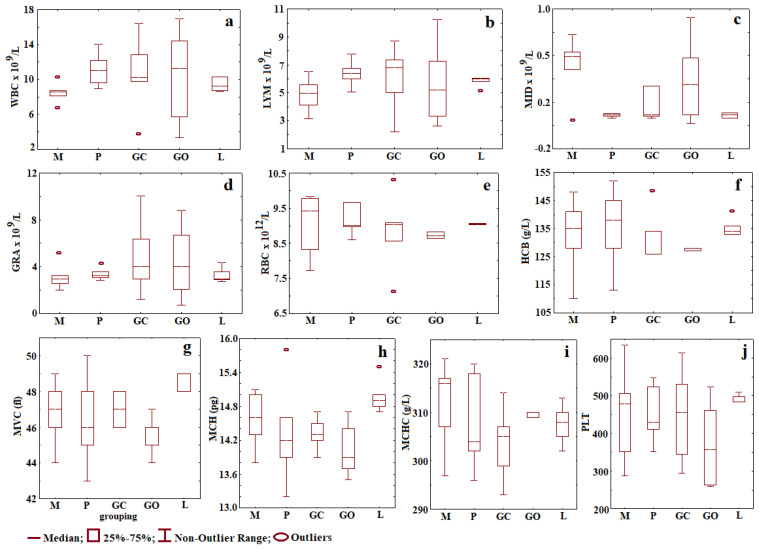
Means and standard deviations for all groups and variables without outliers: (**a**) WBC; (**b**) LYM; (**c**) MID; (**d**) GRA; (**e**) RBC; (**f**) HGB; (**g**) MVC; (**h**) MCH; (**i**) MCHC; (**j**) PLT. (Physiological values: WBC: 4–12 × 10^9^/L, LYM: 2–14.1 × 10^9^/L, MID: 0–0.98 × 10^9^/L, GRA: 0.1–5.4 × 10^9^/L, RBC: 9–15 × 10^12^/L, HGB 90–150 mg/dL, HCT: 24–45%, PLT: 250–750 × 10^9^/L).

A significant increase in the total WBC count in the P group (11.16 ± 2.06) was observed compared to the M group (8.46 ± 1.13) (Figure 9) (*p* < 0.05); however, both recorded values were within the physiological limits of the species (4–12 × 10^9^/L). At the same time, a statistically significant decrease (*p* < 0.05) of the cells mean number (monocytes) in both P (0.13 ± 0.11) and L groups (0.12 ± 0.10) compared to the M group (0.50 ± 0.28) was observed (Figure 9). Nevertheless, the values evidenced by monocytes are within the species’ physical limits (0–0.98 × 10^9^/L).

Encouraging results was obtained in the treatment of some clinical pathologies using photodynamic therapy that involve a photochemical reaction between photosensitizer and laser light [20,21].

The antimicrobial effect of the photodynamic therapy is based on an oxidative explosion due to the light therapy and is based on the deterioration of the cellular structures and of the biomolecules, making, thus, a nonspecific mechanism. aPDT requires the presence of three components: (I) a non-toxic dye, the so-called photosensitizer; (II) visible light of a corresponding wavelength; (III) molecular oxygen. The absorption of light by the PS leads to a transition to its triple state, through which there are two reaction mechanisms to allow the PS to regain its baseline state. In the type I mechanism, the charge is transferred to a substrate or to molecular oxygen generating reactive oxygen species such as hydrogen peroxide, and oxygen radicals such as superoxide ions or free hydroxyl radicals. In the type II mechanism, only energy—not charged—is transferred directly to molecular oxygen, from which simple reactive oxygen (^1^O_2_) comes [22,23,24].

Therefore, combining photosensitizers with light and molecular oxygen species derived from enhanced water could generate a mechanism of cellular death by provoking cytotoxicity to bacterial etiological factors, which are implicated in periodontal disease. 

Major active ingredients of turmeric include three curcuminoids: curcuma (diferuloylmethane, the primary constituent who gives the bright yellow color), desmethoxycurcumin, and bisdemethoxycurcumin, also a volatile oils (turmerone, atlantone and zingiberone), sugars, proteins, and resins [25].

The studies demonstrated that curcuma have important antibacterial, antiinflamatory, antioxidant and anticarcinogenic effects. The anti-infective effects makes curcuma appropriate in the wound-healing. Recent studies on the wound-healing properties of curcuma evidence its ability to increase the granulation tissue formation, collagen deposition, tissue changing structure, and wound reducing, favoring, in this way, the healing process [26,27,28,29,30]. 

Two of the major bioactive constituents, turmerone and atlantone, are presented in turmeric. Curcuma is a hydrophobic photosensitizer that is soluble in *dimethyl sulfoxide* (DMSO), acetone, ethanol, and oils; therefore, it was decided to add arnica oil to the curcuma-based gel. 

Combined with the natural compounds isolated from Arnica Montana oil, a medicinal plant widely used as an herbal remedy containing terpenoids, sesquiterpene lactones, flavonoids, and tannins with anti-inflammatory, antifungal, antimicrobial, and antibiotic properties [31], this could provide a potential mechanism of action highlighting the benefits of our implemented therapy [32]. 

Essential oils of oregano (Origanum vulgare) are widely recognized for their antimicrobial activity, as well as their antiviral and antifungal properties. It is one of the most used aromatic plants, whose essential oils are particularly rich in mono- and sesquiterpenes [33]. In vitro studies of experimental gels with essential oils show anticariogenic and antibiofilm activities [34]. 

Nevertheless, recent investigations have demonstrated that these compounds are also potent antioxidant and anti-inflammatory agents. Carvacrol (CV), the main compound found in essential oils of oregano, is a phenolic monoterpenoid, which possesses a wide range of bioactive properties [35] and has been isolated within our experimental compound. These properties of oregano essential oils are a potential interest to the food, cosmetic, and pharmaceutical industries [36].

According to the literature, curcumin used as photosensitizer activated by blue light in the aPDT procedure has the potential for reducing the bacterial count in periodontal infection. Etemadi et al. states that several researchers have shown that curcuma as a photosensitizer activated by a blue wavelength is effective in the elimination of the various bacterial species involved in periodontal disease [33].

Belinello-Souza et al. found that to the treated animals with aPDT compared with scaling and root planning (SRP) group, the bone gain was approximately 30%, following 7 days after periodontal intervention. [37].

Pre-clinical study on animal models offer important information regarding the treatment methods in investigating the pathogenesis of periodontal disease. In addition to the immunological and microbiological characteristics of rats, the histological features of periodontal collagen fibrils, alveolar bone, cellular cement, connective tissue, junctional epithelium, oral gingival epithelium, and sulcular epithelium are similar to human periodontal tissues [14,38]. This model cannot completely represent all aspects of periodontitis in humans, but is considered an effective method for the exploration of its mechanisms [39], which is why this experimental protocol was chosen.

According to the IACUC standards, body weight is considered an indicator of the health and well-being of laboratory animals. This demonstrates that dental therapy with our materials tested in combination with photodynamic therapy does not negatively affect food intake, apprehension, and mastication. The animals in the treated groups (GO, GC, L) gained approximately 80 g of weight during the 4-week period, whereas those untreated gained only 40 g. Thus, this indicates a variable period of caloric restriction most likely induced by the pain caused by the periodontal disease, as well as dental and gingival mobility and bleeding [40]. According to Toth et al., weight gain is a positive indicator for animal welfare [41].

A complete blood count may reveal general pathological conditions of the body as evidenced by anemia, systemic infections, or blood neoplasm. This is also performed for monitoring a disease or a medical treatment [42].

At the end of the experiment, all values recorded in hematological analysis were within the physiological range of species [43]. This indicates the lack of an inflammatory or infectious chronic systemic process. After 4 weeks, an inflammatory reaction due to periodontitis or local treatment cannot be observed. The lack of adverse systemic hematological reactions is correlated with the clinical symptoms of the rats, having a good maintenance status. Different studies have investigated the effect of aPDT treatment on reducing the inflammation in the gingival tissue of Wistar rats with periodontal disease induced using ligature. Carvalho et al. observed the benefits of aPDT in periodontal disease, due to their immune modulator response in reducing the inflammatory effect and obtaining a resorption of bone tissue [44]. 

Histopathological analysis in this study demonstrated a greater magnitude of inflammatory response and severe destruction of periodontal ligaments in rats who did not receive treatment after ligation removal. It became obvious that ligation was effective in developing experimental periodontal disease. Our experimental protocol is reminiscent of that of Graves et al., which observed that the ligation favors bacterial plaque accumulation, epithelial ulceration, and periodontal tissue invasion by bacteria [45,46].

In the periodontitis group, bone loss due to the osteoclastic activity conferred by the local infections and inflammatory processes was observed. The same bone resorption, but on a smaller scale, was observed in the laser-treated group. 

Remarkably, no bone resorption and minimal inflammatory local reactions were observed in the two experimental photosensitizers groups. However, it is worth mentioning that oregano-based treatment (protocol) has the most beneficial impact based on its capacity to provoke regeneration and the total absence of inflammation.

Knowledge about macro- and micro-structural characteristics of bone tissue may improve the ability to estimate in vivo its quality and quantity. For this purpose, computed tomography (CT) imaging techniques are appropriate and enable assessing the micro-architecture of bone with 2D and 3D quantitative evaluation [47]. CT investigation allows us to quantify different bone parameters, such as geometry, mass, and mineral density, simultaneously. The limitations of the study are: a lack of tests for the group with induced periodontal disease treated only with PS, without light therapy; MTT assay without phototoxicity on the cell lines, and the use of the same laser wavelength for curcuma and oregano, even if oregano absorbs on 370 nm and curcuma on 440 nm.

## 3. Conclusions

This study demonstrates that aPDT was an effective adjuvant treatment of induced periodontal disease in groups treated with natural photosensitizers based on oregano essential oil and curcuma extract. Histopathological analysis suggested the anti-inflammatory effect of aPDT in the presence of natural PS tested in this study.

Periodontal defects in rat mandibles were displayed using CT and compared with histological specimens. The results of histological and computed tomography analyses sustain that the bone loss at the alveolar level obtained through the induced periodontitis, was improved at the end of the treatment.

The cytotoxic effect of new photosensitizers on dental pulp mesenchymal stem cells (dMSCs) and human keratinocyte (HaCaT) in vitro was observed.

In this study, we evidenced the adjuvant effect of natural photosensitizers, based on curcuma extract and oregano essential oil, on induced periodontal disease. The obtained results of these preliminary studies encourage us to continue the research of periodontitis treated with natural photosensitizers activated by photodynamic therapy. 

## 4. Materials and Methods

Newly developed gels based on natural compounds were used as photosensitizers in the aPDT experimental protocol. Natural revealers contain nanocapsules, which include an organic phase based on the essential oil of oregano (Young Living Europe B.V., Groningen, The Netherlands) and curcuma extract with arnica oil, having the active ingredient wrapped in a thin film of polycaprolactone. This technique ensures the controlled release of the active substance through the diffusion phenomenon.

The gels were prepared according to the following proportions: glycerol (Sigma–Aldrich Inc., St. Louis, MO, USA) in a weight ratio of 1:1, 60 mL Kaqun^®^ water (KAQUN Distribution Kft., Nagytarcsa, Hungary), and 0.015% salicylic acid solution. The gels formed were divided into equal parts, in which oregano essential oil (20–50 µg/g according to GSMS analysis) and curcuma extract (70 µg/g according to HPLC Chromatography) were added. 

The **cytotoxicity** assay of oregano essential oil and curcuma extract were performed using human dental pulp mesenchymal stem cells (dMSCs) and human keratinocyte HaCaT cell lines. The cells were cultured according to standard conditions. The potential cytotoxicity of the essential oils was assessed with (4,5-dimethylthiazol-2-yl)-2,5-diphenyltetrazolium bromide (MTT) assay. In order to obtain cell suspensions, the cells were treated with 0.25% trypsin-EDTA, and after centrifugation (1500 rpm for 5 min), 1 × 10^4^ cells/well were seeded on 96-well plates in 200 µL complete culture medium. After 24 h, 0.1 g from each product was tested using a hanging cell culture insert with a pore size of 0.4 µm. Control samples were represented by untreated cells. Each experimental condition was performed in triplicate. Cell proliferation analysis was performed after 24 h. After 24 h, the medium was removed and 100 µL of 1 mg/mL MTT solution (Sigma–Aldrich, St. Louis, MO, USA) was added. After 3 h of incubation at 37 °C in dark, the MTT solution was removed from each well and 150 µL of DMSO (dimethyl sulfoxide) solution (Fluka, Buchs, Switzerland) was added. Spectrophotometric readings at 450 nm were performed with a BioTek Synergy 2 microplate reader (Winooski, VT, USA).

The cell viability of each medium was calculated using the following formula:

(1) $$\%\;\mathrm{Viability}=\left(\frac{\mathrm{Absorbance}\;\mathrm{of}\;\mathrm{samples}}{\mathrm{Absorbance}\;\mathrm{of}\;\mathrm{control}}\right)\times 100$$

### 4.1. Statistical Analysis

The statistical analysis was completed with one-way ANOVA and Tukey Test, using GraphPad Prism version 4.00 for Windows (GraphPad Software, San Diego, CA, USA). The data obtained were presented as the mean of OD540 triplicate measurements ± standard deviation (SD), and a *p* value less than 0.05 was considered statistically significant.

### 4.2. Pre-Clinical Study

This study was performed on 25 adult male Wistar rats (14 months of age) that weighed 420–530 g. Animals were housed in the Establishment for Breeding and Use of Laboratory Animals of USAMV (Cluj-Napoca, Romania) in standard conditions, temperature 22–23 °C, humidity 55%, and 12-h light/dark cycle. The rats were kept in plastic cages with free access to standard rodent granular food (Cantacuzino Institute, Bucharest, Romania) and water ad libitum. The rats were allowed to acclimate to the laboratory environment for a period of 3 weeks. All procedures that involved the use of laboratory animals followed the European guidelines and rules 337, as established by the EU Directive 2010/63/EU and the Romanian law 43/2014 and were preformed by an experienced practitioner. The study protocol was approved by the Research Ethics Committee of the University of Agricultural Sciences and Veterinary Medicine Cluj-Napoca, Romania, and they were authorized by the State Veterinary Authority (aut. No. 52/30.03.2017).

### 4.3. Experimental Design

**Figure 10:**
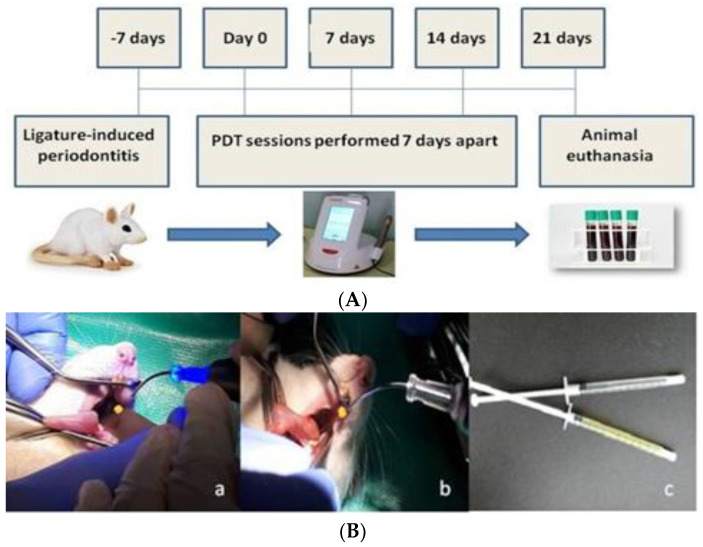
(**A**). The treatment regimen followed in the periodontal disease induced in rats; (**B**) (**a**,**b**) different stages during aPDT procedure; (**c**) oregano and curcuma-based gels used in the study.

In order to investigate the natural photosensitizers, an experimental periodontal disease was induced [48,49]. For all procedures, the rats were anaesthetized with ketamine (60 mg/kg) and xylazine (6 mg/kg), which were administered via intramuscular injection, according to Flecknell et al. [50]. Access to the oral cavity was achieved with a retractor that provided constant opening of the mouth and that held away the cheeks and the tongue. The left-mandibular first molar from each rat, in all surgical groups, was selected to receive a cotton 4.0 ligature in a submarginal position to induce experimental periodontitis. The ligatures were removed after 7 days (Figure 10). Postoperatively, all animals received subcutaneous injections with tramadol (10 mg/bw) in order to achieve the analgesic effect.

Animals were randomly assigned to 5 groups. The groups (*n* = 5) were assigned according to the following treatments applied locally: group 1 was left without surgical intervention representing the control group (M, *n* = 5); group 2 (P, *n* = 5) received surgical intervention and was left untreated; group 3 (GC, *n* = 5) was treated with a curcuma photosensitizer and aPDT; group 4 (GO, *n* = 5) was treated with oregano photosensitizer and aPDT, and group 5 (L, *n* = 5) was treated with laser only. 

*Treatment* was carried out with the use of low-intensity laser SiroLaser Blue (Sirona, 64625 Bensheim, Deutschland) at a wavelength of 445 nm and 200 mW, continuous wave (CW), contact mode, power density 400 mW/cm^2^, using a periodontal tip with 320 µm diameter, attached to the hand piece. In the GC and GO groups, the PS was instilled into the subgingival area with a blunt needle in apical-coronal direction. After 60 s, the PS was rinsed with 1 mL of Kaqun^®^ water (Harghita, Romania) using a graded syringe. Then in GC, GO and L groups, the laser beams were directed into the pockets for 40 s. Irradiation was maintained for 10 s in 4 equidistant sites (2 vestibular and 2 buccal sites). The procedures were repeated one week apart for 4 weeks (Figure 10A). 

All treatments were applied locally following the procedure derived from the standard human treatment and were carried out by a specialist (Figure 10B).

The animals were closely monitored for the entire length of the study, focusing on infection prevention and analgesic therapy. At the end of the experimental study, blood samples were collected so that hematological and biochemical parameters could be determined. Additionally, body weight was closely monitored. The animals were euthanatized after 4 weeks of study by prolonged narcosis followed by cervical dislocation. Then, the left mandible was harvested from each rat for histological analysis. 

### 4.4. Computed Tomography (CT) Analysis

In order to assess the induced periodontal disease and the effect of the applied treatment, bone density was followed on CT images as well as the size of the periodontal space at the treated hemiarchy compared to the opposite hemiarchy. Siemens Somatom Scope (Siemens Medical Solutions USA, Inc., Malvern, PA, USA) at 130 kV and 50 mAs was applied for CT analysis. Three-dimensional (3D) volume viewing and analysis software (RadiAnt Dicom Viewer, Poznań, Poland) were used to visualize and quantify the data obtained. Also a standardized gray-scale value was used to visualize and analyze the mineralized tissues. The mandibles were scanned after the first and last treatment session, on day 0 and day 21, respectively. Bone density was followed on the CT images, as well as the size of the periodontal space at the treated hemiarchy compared to the opposite hemiarchy.

### 4.5. Complete Blood Count and Biochemistry

Complete blood count was determined using the automatic Abacus Junior Vet hematology counter. From the blood tests were determined: white blood cell (WBC), lymphocyte (LYM), minimum inhibitory dilution (MID), granulocytes (GRA), red blood cell (RBC), hemoglobin (HGB), mean corpuscular volume (MCV), mean corpuscular hemoglobin (MCH), mean corpuscular hemoglobin concentration (MCHC), and platelet (PLT).

### 4.6. Histological Analysis

For the histological examination, bone samples from the mandibular lesion site were harvested. For decalcification, after fixation, the mandibula of the animals were kept in a mix of 8% formic and 8% clorhidric acid for 24 h and embedded in paraffin. The samples were fixed in 10% buffered neutral formalin, embedded in paraffin. The sections were made with a high-precision microtome Leica RM 2125 RT, at 5-μm thick, and stained by the hematoxylin–eosin method (HE). The slides were examined under a BX51 Olympus microscope, and images were taken with an Olympus UC 30 digital camera and processed using Olympus Basic Stream software. Sections were examined by an independent observer blinded to the experimental protocol.

### 4.7. Statistical Analysis of the Data

The Student *t*-test, Origine Pro 8 SRO (Origine Lab Corporation 2007, Northampton MA 01060, USA) was performed for the statistical analysis of the findings in this study. An analysis of variance was performed to determine whether there were significant differences between the different test conditions. The significance was evaluated at the level of *p* ≤ 0.05.

## Figures and Tables

**Table 1:** Mean weight change (standard deviation) and weight variability (g) before and after treatment.

Body Weight				
Crt. No.	Groups	Initially (g)	Final (g)	Weight Gain (%)
1.	Control group (M)	452 ± 32.71	551 ± 24.14	21.9
2.	Periodontitis group (P)	491.4 ± 25.47	530.2 ± 29.07	7.85
3.	Curcuma group (GC)	475.6 ± 10.8	570 ± 14.85 **^, ^^^	19.84
4.	Oregano group (GO)	516 ± 17.92	576.4 ± 26.73 *^, ^^^	11.70
5.	Laser group (L)	438 ± 10.74	513.2 ± 27.39	17.16

(Mean ± SD) (*t*-tests, *n* = 5; * *p* < 0.05, ** *p* < 0.005). Compared with group M, (^^ *p* < 0.005).

## Data Availability

Not applicable.

## References

[1] Kömerik N., Nakanishi H., MacRobert A.J., Henderson B., Speight P., Wilson M. (2003). In Vivo Killing of Porphyromonas gingivalis by Toluidine Blue-Mediated Photosensitization in an Animal Model. Antimicrob. Agents Chemother..

[2] Rajapakse P.S., O’Brien-Simpson N.M., Slakeski N., Hoffmann B., Reynolds E.C. (2002). Immunization with the RgpA-Kgp Proteinase-Adhesin Complexes of Porphyromonas gingivalis Protects against Periodontal Bone Loss in the Rat Periodontitis Model. Infect. Immun..

[3] Nessa N., Kobara M., Toba H., Adachi T., Yamamoto T., Kanamura N., Pezzotti G., Nakata T. (2021). Febuxostat Attenuates the Progression of Periodontitis in Rats. Pharmacology.

[4] Prates R.A., Yamada A.M., Suzuki L.C., França C.M., Cai S., Mayer M.P.A., Ribeiro A.C., Ribeiro M.S. (2011). Histomorphometric and Microbiological Assessment of Photodynamic Therapy as an Adjuvant Treatment for Periodontitis: A Short-Term Evaluation of Inflammatory Periodontal Conditions and Bacterial Reduction in a Rat Model. Photomed. Laser Surg..

[5] Graves D.T., Fine D., Teng Y.T.A., Van Dyke T.E., Hajishengallis G. (2008). The use of rodent models to investigate host-bacteria interactions related to periodontal diseases. J. Clin. Periodontol..

[6] Wang H.H., Lee H.M., Raja V., Hou W., Iacono V.J., Scaduto J., Johnson F., Golub L.M., Gu Y. (2019). Enhanced Efficacy of Chemically Modified Curcumin in Experimental Periodontitis: Systemic Implications. J. Exp. Pharmacol..

[7] Birang E., Talebi Ardekani M.R., Rajabzadeh M., Sarmadi G., Birang R., Gutknecht N. (2017). Comparison of Er:YAG Laser andUltrasonic Scaler in the Treatment of Moderate Chronic Periodontitis: A Randomized Clinical Trial. J. Lasers Med. Sci..

[8] Hosseini N., Yazdanpanah S., Saki M., Rezazadeh F., Ghapanchi J., Zomorodian K. (2016). Susceptibility of Candida albicans and Candida dubliniensis to photodynamic therapy using four dyes as the photosensitizer. J. Dent..

[9] Sperandio F., Huang Y.Y., Hamblin M. (2013). Antimicrobial photodynamic therapy to kill Gram-negative bacteria. Recent Pat. Anti-Infect. Drug Discov..

[10] Lara Alves L.V.G., Curylofo-Zotti F.A., Borsatto M.C., de Souza Salvador S.L., Valério R.A., Souza-Gabriel A.E. (2019). Influence of antimicrobial photodynamic therapy in carious lesion. Randomized split-mouth clinical trial in primary molar. Photodiagnosis Photodyn. Ther..

[11] Ishiyama K., Nakamura K., Kano T., Niwano Y. (2016). Bactericidal Action of Photodynamic Antimicrobial Chemotherapy (PACT) with Photosensitizers Used as Plaque-Disclosing Agents against Experimental Biofilm. Biocontrol Sci..

[12] Misba L., Zaidi S., Khan A.U. (2017). A comparison of antibacterial and antibiofilm efficacy of phenothiazinium dyes between Gram positive and Gram negative bacterial biofilm. Photodiagnosis Photodyn. Ther..

[13] Dascalu (Rusu) M.L., Sarosi C., Moldovan M., Badea M.E. (2017). A Study on Revealing Agents in the Context of Photodynamic Therapy in Dental Medicine—A Literature Review. Defect Diffus. Forum.

[14] Dascalu (Rusu) L.M., Moldovan M., Prodan D., Ciotlaus I., Popescu V., Baldea I., Carpa R., Sava S., Chifor R., Badea M.E. (2020). Assessment and Characterization of Some New Photosensitizers for Antimicrobial Photodynamic Therapy (aPDT). Materials.

[15] Mang T.S., Tayal D.P., Baier R. (2012). Photodynamic therapy as an alternative treatment for disinfection of bacteria in oral biofilms. Lasers Surg. Med..

[16] Garcez A.S., Ribeiro M.S., Tegos G.P., Núñez S.C., Jorge A.O.C., Hamblin M.R. (2007). Antimicrobial photodynamic therapy combined with conventional endodontic treatment to eliminate root canal biofilm infection. Lasers Surg. Med..

[17] De Almeida J.M., Theodoro L.H., Bosco A.F., Nagata M.J.H., Oshiiwa M., Garcia V.G. (2008). In vivo effect of photodynamic therapy on periodontal bone loss in dental furcations. J. Periodontol..

[18] Wood S., Nattress B., Kirkham J., Shore R., Brookes S., Griffiths J. (1999). An in vitro study of the use of photodynamic therapy for the treatment of natural oral plaque biofilms formed in vivo. J. Photochem. Photobiol. B Biol..

[19] Ghasemi M., Etemadi A., Nedaei M., Chiniforush N., Pourhajibagher M. (2019). Antimicrobial efficacy of photodynamic therapy using two different light sources on the titanium-adherent biofilms of *Aggregatibacter actinomycetemcomitans*: An in vitro study. Photodiagnosis Photodyn. Ther..

[20] Tokubo L.M., Rosalen P.L., de Cássia Orlandi Sardi J., Freires I.A., Fujimaki M., Umeda J.E. (2018). Antimicrobial effect of photodynamic therapy using erythrosine/methylene blue combination on Streptococcus mutans biofilm. Photodiagnosis Photodyn. Ther..

[21] Fang-Yen C., Gabel C.V., Samuel A.D.T., Bargmann C.I., Avery L. (2012). Laser microsurgery in Caenorhabditis elegans. Methods Cell Biol..

[22] Cieplik F., Tabenski L., Buchalla W., Maisch T. (2014). Antimicrobial photodynamic therapy for inactivation of biofilms formed by oral key pathogens. Front. Microbiol..

[23] Yan S., Huang Q., Song X., Chen Z., Huang M., Zhang J. (2019). A series of photosensitizers with incremental positive electric charges for photodynamic antitumor therapy. RSC Adv..

[24] Yoo J.O., Ha K.S. (2012). New insights into the mechanisms for photodynamic therapy-induced cancer cell death. Int. Rev. Cell. Mol. Biol..

[25] Jurenka J.S. (2009). Anti-inflammatory properties of curcumin, a major constituent of Curcuma longa: A review of preclinical and clinical research. Altern. Med. Rev..

[26] Akbik D., Ghadiri M., Chrzanowski W., Rohanizadeh R. (2014). Curcumin as a wound healing agent. Life Sci..

[27] Mohanty C., Sahoo S.K. (2017). Curcumin and its topical formulations for wound healing applications. Drug Discov. Today.

[28] Zhang Y., McClain S.A., Lee H.M. (2016). A Novel Chemically Modified Curcumin “Normalizes” Wound-Healing in Rats with Experimentally Induced Type I Diabetes: Initial Studies. J. Diabetes Res..

[29] Nasri H., Sahinfard N., Rafieian M., Rafieian S., Shirzad M., Rafieian-Kopaei M. (2014). Turmeric: A spice with multifunctional medicinal properties. J. HerbMed Pharmacol..

[30] Xiao C.J., Yu J., Xie J.L., Liu S., Li S. (2018). Protective effect and related mechanisms of curcumin in rat experimental periodontitis. Head Face Med..

[31] Judžentien A., Būdienė J. (2009). Analysis of the chemical composition of flower essential oils from Arnica montana of Lithuanian origin. Chemija.

[32] Rostro-Alanis M., Báez-González J., Torres-Alvarez C., Parra-Saldívar R., Rodriguez-Rodriguez J., Castillo S. (2019). Chemical Composition and Biological Activities of Oregano Essential Oil and Its Fractions Obtained by Vacuum Distillation. Molecules.

[33] Etemadi A., Hamidain M., Parker S., Chiniforush N. (2021). Blue Light Photodynamic Therapy with Curcumin and Riboflavin in the Management of Periodontitis: A Systematic Review. J. Lasers Med. Sci..

[34] De Oliveira Carvalho I., Aparecida Purgato G., Soares Píccolo M., Ramos Pizziolo V., Ribeiro Coelho R., Diaz-Muñoz G., Alves Nogueira Diaz M. (2020). In vitro anticariogenic and antibiofilm activities of toothpastes formulated with essential oils. Arch. Oral Biol..

[35] Sharifi-Rad M., Varoni E.M., Iriti M., Martore M., Setzer W.N., Contreras M., Salehi B., Soltani-Nejad A., Rajabi S., Tajbakhsh M. (2018). Carvacrol and human health: A comprehensive review. Phytother. Res..

[36] Leyva-López N., Gutiérrez-Grijalva E.P., Vazquez-Olivo G., Heredia J.B. (2017). Essential Oils of Oregano: Biological Activity beyond Their Antimicrobial Properties. Molecules.

[37] Belinello-Souza E.L., Alvarenga L.H., Lima-Leal C., Almeida P., Leite C.G., Lima T.R. (2017). Antimicrobial photodynamic therapy combined to periodontal treatment: Experimental model. Photodiagnosis Photodyn. Ther..

[38] Uslu M.Ö., Eltas A., Marakoğlu I., Dündar S., Şahin K., Özercan I.H. (2018). Effects of diode laser application on inflammation and mpo in periodontal tissues in a rat model. J. Appl. Oral Sci..

[39] Lin P., Niimi H., Ohsugi Y., Tsuchiya Y., Shimohira T., Komatsu K., Liu A., Shiba T., Aoki A., Iwata T. (2021). Application of Ligature-Induced Periodontitis in Mice to Explore the Molecular Mechanism of Periodontal Disease. Int. J. Mol. Sci..

[40] Fernandes L.A., Theodoro L.H., Martins T.M., de Almeida J.M., Garcia V.G. (2010). *J* Effects of diode laser application on inflammation and mpo in periodontal tissues in a rat model. Appl. Oral Sci..

[41] Robinson M., Hart D., Pigott G.H. (1991). The effects of diet on the incidence of periodontitis in rats. Lab. Anim..

[42] Toth L.A., Gardiner T.W. (2000). Food and water restriction protocols: Physiological and behavioral considerations. Contemp. Top. Lab. Anim. Sci..

[43] Smith C., Jarecki A. (2013). Atlas of Comparative Diagnostic and Experimental Hematology.

[44] Giknis M.L.A., Charles B., Clifford D.V.M. (2008). Clinical Laboratory Parameters for Crl:CD(SD) Rats.

[45] Carvalho A.S., Napimoga M.H., Coelho-Campos J., Silva-Filho V.J., Thedei G. (2011). Photodynamic therapy reduces bone resorption and decreases inflammatory response in an experimental rat periodontal model. Photomed. Laser Surg..

[46] Graves D.T., Kang J., Andriankaja O., Wada K., Rossa C. (2012). Animal models to study host-bacteria interactions involved in periodontitis. Front. Oral Biol..

[47] Faot F., de Camargos G C., Duyck J., Vandamme K. (2015). Micro-CT analysis of the rodent jaw bone micro-architecture: A systematic review. Bone Rep..

[48] Garcia V.G., Knoll L.R., Longo M., Novaes V.C.N., Assem N.Z., Ervolino E. (2016). Effect of the probiotic Saccharomyces cerevisiae on ligature-induced periodontitis in rats. J. Periodontal Res..

[49] Garcia V.G., Erivan C.G.V., Fernandes L.A., Bosco A.F., Nagata H.M., Casatti A.C., Ervolino E., Theodoro L.H. (2013). Adjunctive antimicrobial photodynamic treatment of experimentally induced periodontitis in rats with ovariectomy. J. Periodontol..

[50] Flecknell P. (2009). Laboratory Animal Anaesthesia.

